# Cell cycle arrest and apoptosis induction by methanolic leaves extracts of four Annonaceae plants

**DOI:** 10.1186/s12906-017-1811-3

**Published:** 2017-06-05

**Authors:** Kitti Pumiputavon, Tanawat Chaowasku, Chalermpong Saenjum, Maslin Osathanunkul, Boonsong Wungsintaweekul, Kriangkrai Chawansuntati, Jiraprapa Wipasa, Pathrapol Lithanatudom

**Affiliations:** 1 Department of Biology, Faculty of Science, Chiang Mai University, Chiang Mai, 50200 Thailand; 2 Department of Pharmaceutical Science, Faculty of Pharmacy, Chiang Mai University, Chiang Mai, 50200 Thailand; 3 School of Pharmacy, Walailak University, Nakhon Si Thammarat, 80160 Thailand; 4 Research Institute for Health Sciences, Chiang Mai University, Chiang Mai, 50200 Thailand

**Keywords:** Annonaceae, Anti-cancer activity, Cell cycle arrest, Apoptosis, Quercetin, Rutin

## Abstract

**Background:**

*Uvaria longipes* (Craib) L.L.Zhou, Y.C.F.Su & R.M.K.Saunders, *Artabotrys burmanicus* A.DC, *Marsypopetalum modestum* (Pierre) B.Xue & R.M.K.Saunders and *Dasymaschalon* sp. have been used for traditional medicine to treat cancer-like symptoms in some ethnic groups of Thailand and Laos.

**Methods:**

We evaluated the anti-cancer activity of these Annonaceae plants against several human cancer cell lines. The apoptosis induction was detected by Annexin/propidium iodide (PI) staining. Phytochemical screening was tested by standard protocols and bioactive compounds were determined by HPLC.

**Results:**

The crude extracts from leaves of *U. longipes*, *Dasymaschalon* sp., *A. burmanicus*, and *M. modestum* showed particular effects that were found to vary depending on the cancer cell line, suggesting that the effect was in a cell-type specific manner. Interestingly, the induction of apoptotic cell death was prominent by the leaves-derived crude extract of *M. modestum*. This crude was, therefore, subjected to cell cycle analysis by PI staining. Results showed that this crude extract arrested cell cycle and increased the percentage of cells in the SubG1 phase in some cancer cell lines. The phytochemical screening tests indicated that all crude extracts contained tannins and flavonoids. HPLC of flavonoids using standards identified rutin as an active compound in *U. longipes* and *Dasymaschalon* sp., whereas quercetin was found in *U. longipes* and *M. modestum*.

**Conclusions:**

These crude extracts provide a new source for rutin and quercetin, which might be capable of inducing cancer cell apoptotic death in a cell-type specific manner. This suggests, by analyzing the major bioactive compounds, the potential use of these crudes for chemotherapy in the future.

## Background

Cancer refers to a group of cells with growth disorder arising from aberrant cell division. The resulting phenotypes involve uncontrollable cell growth with the possibility of the disorder spreading throughout the body [1]. To date, more than 100 types of cancer have been reported [2]. A number of studies have shown that cancer is one of the primary causes of death worldwide, second only to cardiovascular disorders [3]. Furthermore, morbidity and mortality associated with cancer are expected to increase annually, thus becoming a serious global public health problem.

Accumulating evidence suggests that development of cancer is mostly triggered by external factors or environmental factors (90%). These include smoking (30%), diet and obesity (35%), infection (20%), radiation (10%), and stress and environmental pollution (5%), while the genetic factor contributes only 10% to the cause of cancer [1]. Definitive cancer treatment is crucial and urgently required for future management of the disease. Traditional remedial methods of cancer, comprising surgery, chemotherapy, and radiotherapy, although proven to be effective, have several major drawbacks such as detrimental side effects, cancer resistance, and relapses [4]. Hence, acquisition of novel alternative cancer treatment methods is necessary.

Induction of apoptosis, a programmed cell death mechanism for wiping out unwanted cells in tissue, is one of the effective strategies to kill cancer cells [5]. This procedure is mainly associated with morphological change, heterochromatin condensation, cell shrinkage and budding, loss of organelles in the cytoplasm, and formation of apoptotic bodies [6]. Apoptosis can be induced through extrinsic and/or intrinsic pathways. The extrinsic pathway is initiated by the interaction of death receptors and specific signaling molecules, while the intrinsic pathway is primarily stimulated by cellular sensing of extracellular and/or intracellular stresses, both of which require appropriate stimuli to trigger [7]. Therefore, continual pursuit of natural compounds having the ability to trigger apoptosis pathways in cancer cells is currently gathering much interest and becoming more attractive in the field of oncology [8].

The Annonaceae is a tropical plant family of trees, shrubs, and lianas. There are 109 validly described and recognized genera and approximately 2440 species in this family [9]. Interestingly, numerous bioactive compounds have been isolated from the Annonaceae [10]. Alkaloids are among the most important natural compounds of the Annonaceae family. A previous study has reported the isolation of isoquinoline alkaloids from this family [11]. Additionally, terpenoids [12] and acetogenins [13] can also be isolated from some species of the Annonaceae family. Certain types of alkaloids, such as jerantinine B [14], liriodenine [15], and vinoreline [16], exhibit the ability to induce apoptosis and block the cell cycle in the G1 phase. Moreover, rutin and squamocin B were reported as bioactive flavonoids in *Annona squamosal* [17]. There are plenty of species from the Annonaceae waiting to be studied with the potential to uncover novel anti-cancer bioactivity compounds.

In this study, we used three groups of cancer cell lines representing the three major cancer types observed with significantly high incidence worldwide, including human cervical carcinoma, human hepatocellular carcinoma, and human hematopoietic cell lines, as in vitro experimental models to evaluate the anti-cancer activity of four genera (four species) of the Annonaceae, viz. *Uvaria longipes* (Craib) L.L.Zhou, Y.C.F.Su & R.M.K.Saunders, *Dasymaschalon* sp., *Artabotrys burmanicus* A.DC.*,* and *Marsypopetalum modestum* (Pierre) B.Xue & R.M.K.Saunders. These species were chosen because there has been no previous study on anti-cancer activity, but certain species in the genus *Uvaria* L. [18, 19], *Dasymaschalon* Dalla Torre & Harms [20, 21], and *Artabotrys* R.Br. [22] had been shown to exhibit considerable anti-cancer activities. High performance liquid chromatography (HPLC) was also performed to identify bioactive components in all studied crude extracts.

## Methods

### Chemicals

RPMI (Roswell Park Memorial Institute) medium, FBS (fetal bovine serum), Penicillin–Streptomycin, L-Glutamine, Fungizone, and 0.25% Trypsin-EDTA were purchased from Gibco-BRL, USA. Annexin V-FITC (fluorescein isothiocyanate) was purchased from ImmunoTools GmbH, Germany. HEPES was purchased from Merck Millipore, Germany. Sodium chloride, sodium bicarbonate, and calcium chloride were purchased from RCI LABSCAN, Thailand. Dimethyl sulfoxide (DMSO), Triton X-100, and propidium iodide (PI) were purchased from Sigma-Aldrich, USA. Ribonuclease A (RNase A) was purchased from Worthington Biological Corporation, USA. All solvents and chemicals used were either HPLC grade or analytical grade and were purchased commercially from Sigma Chemical Co. (St. Louis, MO), Fluka Chemical Co. (Switzerland), and Merck (Darmstadt, Germany).

### Cell lines and culture

The human cancer cell lines used in this study consisted of human cervical carcinoma (HeLa, SiHa, and CaSki) (a kind gift from Assoc. Prof. Tipaya Ekalaksananan, Khon Kaen University, Thailand), human hepatocellular carcinoma (HepG2 and Hep3B) (a kind gift from Prof. Duncan R. Smith, Mahidol University, Thailand), and human myeloid leukemia (K562, U937, and RAJI) (a kind gift from Prof. Sumalee Tungpradabkul, Mahidol University, Thailand). All the cell lines were maintained in the RPMI medium containing 10 mM of HEPES, 1 mM of sodium bicarbonate, 10% fetal bovine serum (FBS), penicillin (100 IU/ml), and streptomycin (100 μg/ml) (RPMI complete media) at 37 °C in a humidified 5% CO_2_ atmosphere.

### Plant materials

*Uvaria longipes* (collection no.: Chaowasku 132), *Dasymaschalon* sp. (collection no.: Chaowasku 120), and *Marsypopetalum modestum* (collection no.: Chaowasku 164) were collected from private residences at coordinates 13.790384, 100.372378; and *Artabotrys burmanicus* (collection no.: Chaowasku 163) was collected from a private garden at coordinates 13.919300, 99.952555. All the voucher specimens were deposited in the Chiang Mai University Biology (CMUB) herbarium. It should be noted that the *Dasymaschalon* sp*.* used in our study is roughly identified as *Dasymaschalon lomentaceum* and is currently authenticated for its potentially new species as evidenced by both morphological and molecular data (manuscript in preparation). The collection, preparation and identification of all plant specimens used in this study was performed by Dr. Tanawat Chaowasku.

### Preparation of crude extracts

Leaves of these plants were washed, air dried at 25-30 °C and crushed to powder. Dried and powdered leaves of *U. longipes*, *Dasymaschalon* sp., *A. burmanicus* and *M. modestum* were incubated in methanol at room temperature for 24 h. Then, the solution was collected and evaporated under vacuum in a rotary evaporator. All the methanolic extracts were dissolved in dimethyl sulfoxide (DMSO) at a concentration of 100 mg/ml. Finally, these extracts were serially diluted into 1000 μg/ml, 500 μg/ml, 250 μg/ml, and 125 μg/ml in RPMI complete media.

### Annexin V staining assay

Human cancer cell lines (HeLa, SiHa, CaSki, HepG2, Hep3B, K562, U937, and RAJI) at 1 × 10^5^ cells/ml were cultured in 24-well plates in the presence of various concentrations of methanolic extracts (1000 μg/ml, 500 μg/ml, 250 μg/ml, and 125 μg/ml) for 24 h. The quantification of the apoptotic cells was measured by Annexin V-FITC (fluorescein isothiocyanate)/PI (propidium iodide) co-staining assay. Briefly, at the end of the 24 h incubation, the cells were harvested and centrifuged at 1800 rpm for 8 min. The pellet was resuspended in 50 μl binding buffer containing 0.5 μl Annexin V-FITC and then incubated at 4 °C for 30 min in the dark. PI (50 μg/ml) in 200 μl binding buffer was added to each of the tubes and incubated for 5 min. Finally, the cells were analyzed by flow cytometry (CyAn ADP Analyzer, Beckman Coulter, USA).

### Cell cycle analysis

Human cancer cell lines at 1 × 10^6^ cells were cultured in 6-well plates in the presence of leaves of *M. modestum* methanolic extracts (1000 μg/ml, 500 μg/ml, 250 μg/ml, and 125 μg/ml) for 24 h. After treatment, the cells were washed and centrifuged at 1800 rpm for 8 min. The cells were resuspended and fixed with 70% ethanol at 4 °C for 2 h. After fixing, the cells were washed with PBS and centrifuged at 1800 rpm for 8 min. The pellet was broken up by vortexing and then resuspended in 250 μl PBS containing PI (20 μg/ml), RNase A (20 μg/ml), and Triton X-100 (0.1%), and incubated for further 30 min in the dark. Finally, the cells were analyzed by flow cytometry (BD FACSCalibur, BD Biosciences, USA).

### Phytochemical screening tests

The four crude leaf extracts were tested for constituents such as alkaloids, sterols, cardiac glycosides, anthaquinone glycosides, saponins, flavonoids, and tannins by using standard methods as described previously [23–25]. The qualitative results are expressed as (−) for the absence and (+) for the presence of constituents.

### Determination of rutin and quercetin by reversed-phase HPLC

The content of quercetin and rutin in the four crude extracts was determined by using a reversed-phase HPLC (RP-HPLC) system (Shimadzu Corporation, Japan) including LC-10AV *VP* pumps and SPD-10AV *VP* with UV detector. The column for the separation was 250 × 4.6 mm in diameter (SymmetryShield® RP18 C18; Water Co., Ltd.). The mobile phases used for the determination of quercetin and rutin consisted of 5 mM KHPO_4_:acetonitrile:methanol in the ratio of 49:40:11% *v*/v and de-ionized H_2_O:Methanol:Triethylamine in the ratio of 60:40:0.1, respectively. The flow rate and the detection wavelengths for quercetin and rutin were 0.7 and 0.5 mL/min, and 350 and 256 nm, respectively. All the crude extracts were subjected to the RP-HPLC system in parallel with known concentrations of quercetin dihydrate and rutin (GmBH, Germany). The concentrations of quercetin and rutin were calculated from the peak area using the calibration curves. The assays were performed in triplicates.

### Statistical analysis

Statistical analysis was performed using statistical analysis program (SPSS, 16.0, International Business Machines, USA). Comparisons between groups (controls and treatments) were performed by one-way ANOVA with Tukey’s HSD post hoc test. Statistical significance was accepted at *P* value lower than 0.05.

## Results

### Apoptosis assessment

**Fig. 1:**
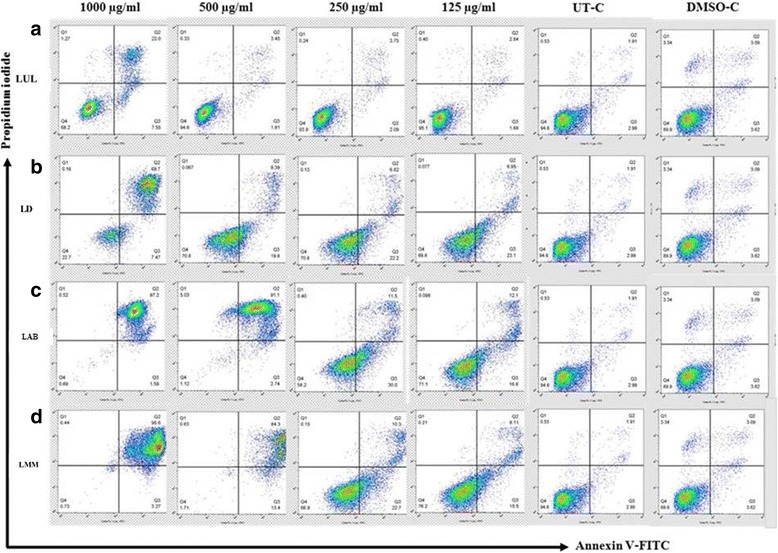
Effect of crude extracts from the leaves of *U. longipes* **a**, *Dasymaschalon* sp. **b**, *A. burmanicus* **c**, and *M. modestum* **d** on the induction of apoptosis in the sample RAJI cell. RAJI cells were cultured in the presence of various concentrations of the crude extracts for 24 h. The cells were then stained with Annexin V and PI, and analyzed by flow cytometry

To investigate the anti-cancer activity of the crude extracts on eight human cancer cell lines, we employed Annexin V and PI co-staining to examine possible induction of cell death (necrosis and/or apoptosis). The percentages of cells analyzed by flow cytometry following Annexin V and PI staining could be classified into four categories. The populations of cells residing in the Annexin V+/PI- and the Annexin V+/PI+ quadrants were determined as early and late apoptotic cells, respectively. The Annexin V−/PI - and the Annexin V−/PI+ quadrants were determined as living cells and necrotic cells, respectively. Both the untreated control (UT-C) and the DMSO-treated cells (DMSO-C) showed the percentages of the apoptotic cells as ranging from 3% to 12% after 24 h of incubation (Table 1). All treatments of crude extracts (leaves from *Uvaria longipes*: LUL, *Dasymaschalon* sp.: LD, *Artabotrys burmanicus*: LAB, and *Marsypopetalum modestum*: LMM) showed varying degrees of percentages of apoptotic cells (the percentages of the early apoptotic cells and the late apoptotic cells combined) depending on the type of crude extract. Consistently, changes in cell morphology were observed under an inverted microscope. This was corroborated by the decreases in the intensity of the forward light scatter when analyzed by flow cytometry (data not shown). Interestingly, the apoptotic effects from individual crude extracts exhibited activity in a cell-type specific manner. For example, the percentages of apoptotic HepG2 cells treated with LUL were significantly higher than those of SiHa cells treated with the same extract (Table 1). A dose-dependent manner was also observed in some crude extracts in particular cell types, for example, RAJI cells treated with LAB and LMM (Table 1 and Fig. 1). In our study, the most prominent apoptotic effect on cancer cell lines was observed in LMM and LAB crude extracts (Table 1), particularly against the RAJI cell line (Fig. 1). The half maximal inhibitory concentrations (IC50) of all crude extracts on cell death are shown in Table 2.

**Table 1:** The average (±SD) percentages of apoptotic cells identified from the dot plots of Annexin V vs PI with statistical analysis

	Concentrations of crude extracts (μg/ml)				Controls	
	1000	500	250	125	UT-C^a^	DMSO-C^b^
HeLa						
LUL^c^	50.9 ± 4.8**	45.3 ± 1.7**	37.3 ± 3.7**	16.2 ± 6.8**	3.1 ± 1.5	3.2 ± 1.3
LD^d^	22.8 ± 1.6**	22.9 ± 1.7**	22.1 ± 0.0**	17.9 ± 0.5**		
LAB^e^	13.4 ± 2.1**	6.0 ± 1.0**	5.9 ± 2.7*	6.6 ± 1.9**		
LMM^f^	70.2 ± 5.0**	33.7 ± 6.9**	2.8 ± 0.4	2.1 ± 0.1		
SiHa						
LUL^c^	17.0 ± 1.3**	30.0 ± 2.0**	18.8 ± 1.3**	7.9 ± 0.0*	5.2 ± 1.7	8.6 ± 1.4
LD^d^	93.1 ± 1.3**	77.1 ± 2.1**	19.2 ± 0.1**	3.2 ± 0.4**		
LAB^e^	87.9 ± 4.8**	84.5 ± 3.7**	37.2 ± 6.2**	4.5 ± 0.4**		
LMM^f^	96.6 ± 0.1**	15.3 ± 1.5**	3.5 ± 1.1**	3.9 ± 0.2**		
CaSki						
LUL^c^	37.5 ± 1.5**	16.1 ± 3.7**	20.6 ± 0.4**	6.0 ± 0.8	6.0 ± 0.2	6.4 ± 1.4
LD^d^	88.2 ± 1.8**	77.9 ± 2.2**	51.9 ± 4.4**	7.6 ± 0.0		
LAB^e^	89.5 ± 4.6**	51.9 ± 3.1**	38.0 ± 1.0**	3.9 ± 0.6**		
LMM^f^	76.1 ± 2.6**	34.4 ± 0.8**	5.6 ± 0.4	7.0 ± 0.9		
HepG2						
LUL^c^	87.1 ± 4.7**	65.2 ± 1.1**	36.7 ± 2.2**	38.9 ± 2.4**	11.1 ± 6.6	12.4 ± 6.6
LD^d^	84.6 ± 3.8**	75.1 ± 1.9**	45.0 ± 0.3**	42.8 ± 3.8**		
LAB^e^	80.1 ± 2.4**	70.5 ± 4.6**	76.7 ± 0.4**	16.4 ± 0.4		
LMM^f^	93.7 ± 2.1**	56.5 ± 4.0**	46.1 ± 7.3**	40.2 ± 2.6**		
Hep3B						
LUL^c^	51.1 ± 0.1**	27.3 ± 2.0**	12.0 ± 0.9**	10.5 ± 1.0**	8.1 ± 0.6	6.9 ± 0.9
LD^d^	43.1 ± 2.5**	13.3 ± 7.9*	7.5 ± 2.6	5.9 ± 0.3		
LAB^e^	54.2 ± 2.3**	57.6 ± 3.6**	43.0 ± 1.3**	36.8 ± 1.3**		
LMM^f^	45.6 ± 6.9**	14.0 ± 2.2**	7.0 ± 8.6	9.1 ± 8.4		
K562						
LUL^c^	44.2 ± 0.7**	8.2 ± 0.0**	6.7 ± 1.1	6.8 ± 0.9	5.6 ± 1.3	5.0 ± 0.1
LD^d^	25.9 ± 4.2**	7.4 ± 0.7**	7.5 ± 2.1	5.6 ± 0.1		
LAB^e^	77.1 ± 6.6**	37.3 ± 3.2**	22.9 ± 1.7**	7.3 ± 1.6		
LMM^f^	79.6 ± 1.3**	13.5 ± 1.3**	5.7 ± 0.6	5.5 ± 0.7		
U937						
LUL^c^	94.2 ± 0.9**	10.1 ± 0.0**	6.0 ± 1.7	6.2 ± 0.0	7.4 ± 1.4	5.7 ± 0.7
LD^d^	88.8 ± 0.3**	8.8 ± 0.4*	8.0 ± 0.3	7.4 ± 1.2		
LAB^e^	29.1 ± 4.1**	8.6 ± 4.2*	5.4 ± 0.9	6.2 ± 0.2		
LMM^f^	84.6 ± 3.0**	54.9 ± 1.2**	5.3 ± 4.9	5.0 ± 3.7		
RAJI						
LUL^c^	71.8 ± 3.4**	32.6 ± 1.6**	29.9 ± 2.6**	23.8 ± 0.5**	5.6 ± 2.2	7.0 ± 3.1
LD^d^	81.0 ± 2.9**	26.9 ± 0.3**	21.1 ± 0.2**	19.4 ± 2.2**		
LAB^e^	98.6 ± 0.1**	93.3 ± 0.6**	41.1 ± 0.4**	24.5 ± 6.1**		
LMM^f^	98.5 ± 0.3**	88.5 ± 3.0**	33.7 ± 1.0**	25.3 ± 2.4**		

Symbols * and ** represent statistically significant differences less than 0.05 and 0.01, respectively, when compared to DMSO-treated control

^a^Untreated control

^b^DMSO-treated control

^c^Crude extract from leaf of *U. longipes*

^d^Crude extract from leaf of *Dasymaschalon* sp

^e^Crude extract from leaf of *A. burmanicus*

^f^Crude extract from leaf of *M. modestum*

**Table 2:** IC_50_ values of leaf crude extracts against cancer cell lines

	IC_50_ value (μg/ml)							
	HeLa	SiHa	CaSki	HepG2	Hep3B	K562	U937	RAJI
LUL^a^	661.16	2985.13	1219.53	386.32	394.94	1262.15	620.56	621.21
LD^b^	1370.02	590.28	465.71	329.88	1125.20	2364.85	1720.44	645.34
LAB^c^	796.46	370.50	298.29	79.15	16.45	419.86	441.49	329.83
LMM^d^	445.91	619.31	669.85	358.68	871.80	726.92	716.84	362.51

^a^Crude extract from leaf of *U. longipes.*

^b^Crude extract from leaf of *Dasymaschalon* sp

^c^Crude extract from leaf of *A. burmanicus.*

^d^Crude extract from leaf of *M. modestum.*

Table 1. Cancer cell lines were incubated for 24 h in the presence of 125–1000 μg/ml of crude extracts (leaf of *U. longipes*: LUL, leaf of *Dasymaschalon* sp*.*: LD, leaf of *A. burmanicus*: LAB, and leaf of *M. modestum*: LMM). Untreated cells (UT-C) and DMSO-treated cells (DMSO-C) were used as the controls. Statistical comparisons were analyzed by the one-way ANOVA with Tukey’s HSD post hoc test.

Table 2. IC_50_ values of leaf crude extract against cancer cell lines was calculated from all types of cell death (Annexin V+/PI-, Annexin V−/PI+ and the Annexin V+/PI+ quadrants).

### Effect of LMM on cell cycle analysis

**Fig. 2:**
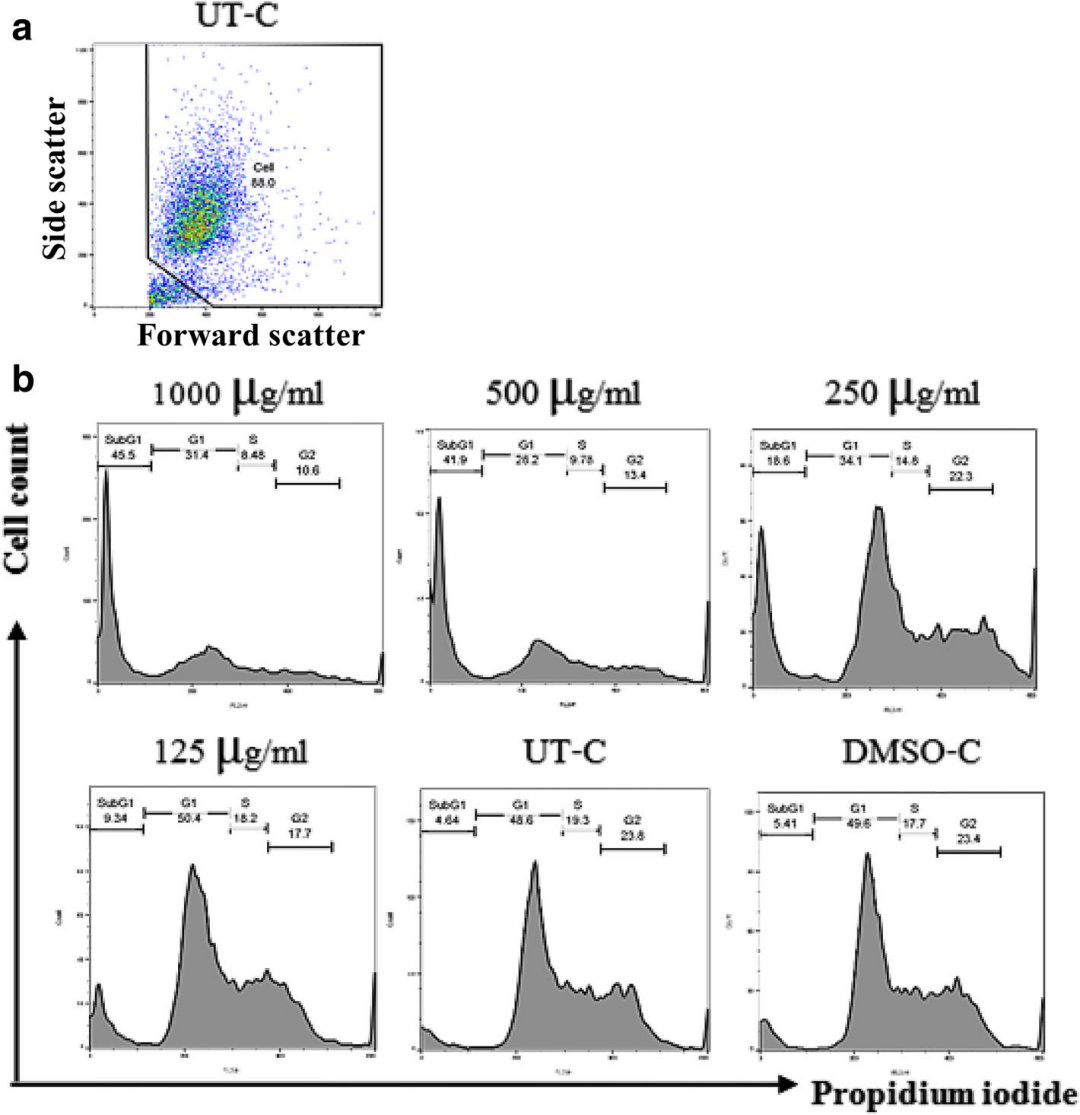
Cell cycle analysis of RAJI cells treated with 125–1000 μg/ml of crude extract from the leaves of *M. modestum* (LMM). Untreated (UT-C) cells and DMSO were used as the controls. **a** shows the gating strategy and **b** histograms show the patterns of cells stained with PI

To test whether the apoptotic cell death was due to the inhibitory effect of the crude extracts on the cell cycle process, cell cycle analysis was then carried out. Although both LMM and LAB showed maximum potent effect on most of the cancer cell lines, LMM was chosen for the cell cycle analysis since there have been no reports on its anti-cancer activity, whereas the crude extract from *A. siamensis* (closely related to A. *burmanicus*) was reported having inhibitory effect on three other cell lines [26]. Furthermore, *A. burmanicus* is a rare species in the genus *Artabotrys* and resources for raw material were limited, making it difficult to study for therapeutic uses in the long run. Cancer cell lines were treated with various concentrations of LMM for 24 h, stained with propidium iodide, and subsequently analyzed by flow cytometry. All the cancer cell lines except for SiHa and Hep3B showed an increase in the DNA fragmentation in a dose-dependent manner as reflected by the increase in the percentages of cells in the sub G1 phase compared to the untreated or the DMSO-treated cells (Table 3 and Fig. 2).

**Table 3:** The average (±SD) percentages of cells in the sub-G1 phase obtained from the histogram of PI staining with statistical analysis

	Concentration of crude extracts (μg/ml)				Controls	
	1000	500	250	125	UT-C^a^	DMSO-C^b^
HeLa	11.2 ± 1.4**	4.0 ± 0.0	3.9 ± 0.3	4.8 ± 0.1	4.0 ± 1.2	6.2 ± 2.7
SiHa	3.5 ± 0.5**	2.4 ± 0.1**	1.0 ± 0.0	1.0 ± 0.3	0.6 ± 0.0	0.7 ± 0.0
CaSki	46.7 ± 0.5**	11.3 ± 1.2**	10.0 ± 1.8**	8.5 ± 1.2	5.6 ± 1.3	6.6 ± 0.5
HepG2	17.2 ± 1.0**	12.6 ± 1.7**	6.9 ± 1.6**	7.1 ± 1.9**	6.2 ± 1.6	2.8 ± 0.7
Hep3B	7.9 ± 1.6**	6.5 ± 1.1*	5.3 ± 0.0*	6.5 ± 0.4*	4.2 ± 4.2	5.4 ± 0.8
K562	46.7 ± 0.5**	11.3 ± 1.2**	10.0 ± 1.8**	8.5 ± 1.2	5.6 ± 1.3	6.6 ± 0.5
U937	55.5 ± 2.4**	10.5 ± 0.5**	5.4 ± 0.1**	3.3 ± 0.1**	1.4 ± 0.0	1.4 ± 0.0
RAJI	43.9 ± 1.6**	41.4 ± 1.3**	15.0 ± 0.3**	8.0 ± 0.2**	4.4 ± 0.0	4.1 ± 0.3

Symbols * and ** represent statistically significant differences less than 0.05 and 0.01, respectively, when compared to DMSO-treated control

^a^Untreated control

^b^DMSO-treated control

Table 3. Cancer cell lines were cultured in the presence of 125–1000 μg/ml of crude extract from the leaves of *M. modestum* for 24 h, stained with PI, and then analyzed by flow cytometry. Statistical comparisons were analyzed by the one-way ANOVA with Tukey’s HSD post hoc test.

### Phytochemical screening

All four leave-derived crude extracts were tested for phytochemicals. The results showed the presence of tannins and flavonoids in all the crude extracts (Table 4). Alkaloids were found in *U. longipes* and *A. burmanicus*, and saponins were found in *U. longipes* and *Dasymaschalon* sp. The phytochemicals in the Annonaceae family observed in our study was consistent with that in a previous report [27].

**Table 4:** Phytochemical analysis of Crude Methanolic Extract

Biochemicals	*U. longipes*	*Dasymaschalon* sp.	*A. burmanicus*	*M. modestum*
Alkaloids	+	−	+	−
Sterols	−	−	−	−
Tannins	+	+	+	+
Saponins	+	+	−	−
Anthraquinone glycosides	−	−	−	−
Cardiac glycosides	−	−	−	−
Flavonoids	+	+	+	+
Coumarins	−	−	−	−

*Note:* (−): Absence; (+): Presence

### High-performance liquid chromatography (HPLC)

**Fig. 3:**
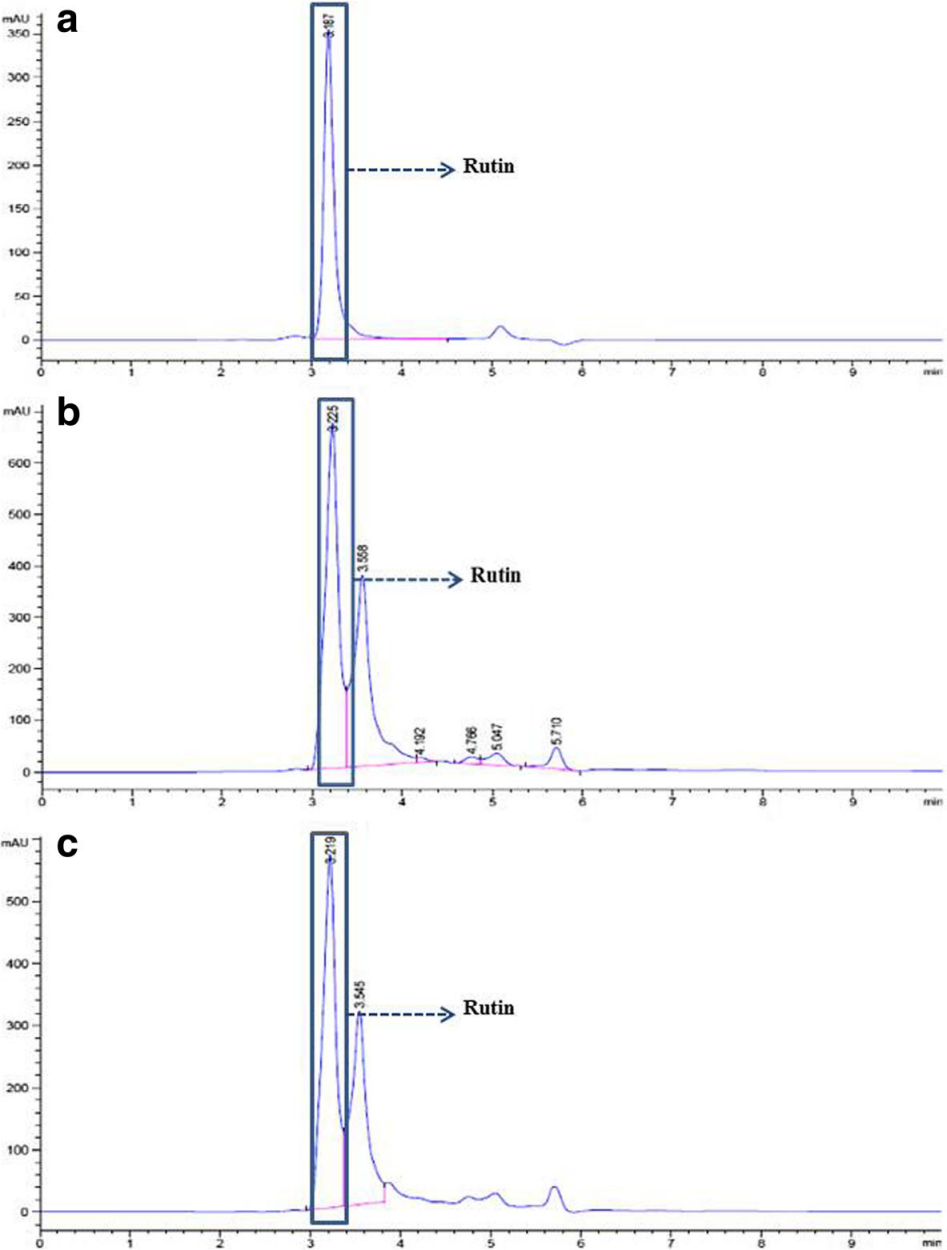
HPLC chromatogram of rutin **a**, crude extracts of *Uvaria longipes* **b** and *Dasymaschalon* sp. (**c**). Arrows show the peaks corresponding to the standard rutin

**Fig. 4:**
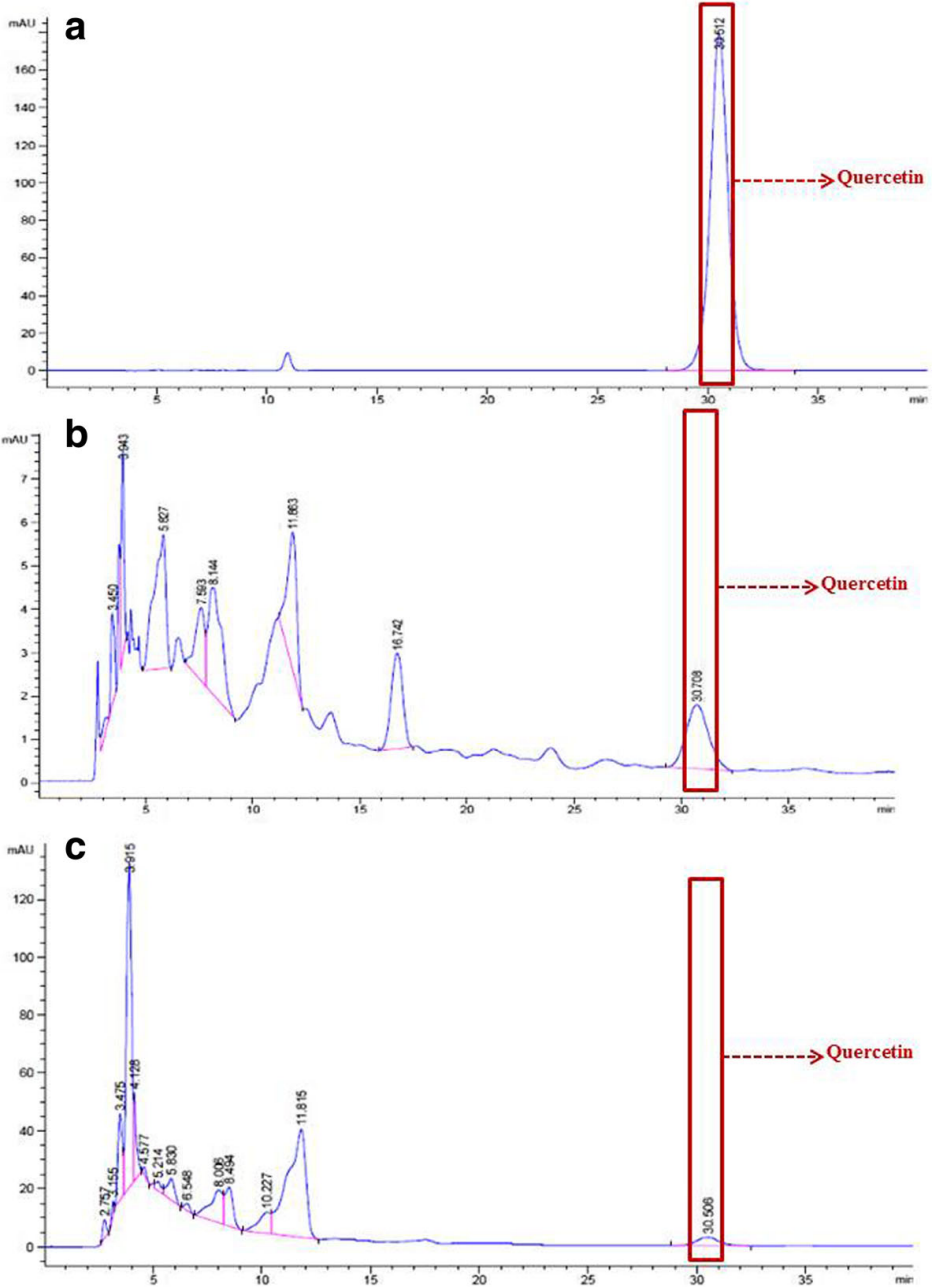
HPLC chromatogram of quercetin **a**, crude extracts of *Uvaria longipes* **b** and *Marsypopetalum modestum* **c**. Arrows show the peaks corresponding to the standard quercetin

Quercetin and rutin have been reported for their numerous biological activities, such as anti-inflammatory, free radical scavenging, immunomodulatory, and cancer chemoprevention [28, 29]. Interestingly, quercetin and rutin have been reported as present in a number of species of the Annonaceae family [30]. Therefore, all the methanolic leaves extracts were analyzed for quercetin and rutin by reversed-phase HPLC. The HPLC chromatogram demonstrated that the leaf methanolic extracts of *U. longipes* (Fig. 3b) and *Dasymaschalon* sp. (Fig. 3c) contain rutin amounting to 3.29 ± 0.22 mg/g extract and 2.05 ± 0.10 mg/g extract, respectively. While, the leaf methanolic extract of *U. longipes and M. modestum* contained quercetin amounting to 1.52 ± 0.13 mg/g extract and 0.43 ± 0.11 mg/g extract, respectively (Fig. 4b and c).

## Discussion

Medicinal plants are plants that can synthesize certain chemical compounds and produce biological activities that protect the plants from insects, fungi, and other herbivores. For a long time in history, human beings have been utilizing medicinal plants for curing a number of diseases [31]. Although revolutions in the modern drug industry have given rise to a trend of replacing herbal remedies with modern drugs, studies of natural compounds from herbal plants and their activities are still of interest as far as discovering novel drugs is concerned. According to a WHO report (2002), 60% of medicinal drugs are isolated from natural sources, including anti-cancer drugs [32].

In this study, we evaluated the anti-cancer activity of leaf methanolic extracts of *U. longipes*, *Dasymaschalon* sp., *A. burmanicus,* and *M. modestum*. These plants belong to the Annonaceae family, a family of particular interest for its secondary metabolites [33]. All selected species have never been reported for any therapeutic application in cancer treatment. Interestingly, *M. modestum,* locally known as the Lao traditional herb, has been shown recently to possess anti-tuberculosis activity in vitro [34]. We evaluated the anti-cancer activity of these four species in three groups of cancer cell lines, including human cervical carcinoma (HeLa, SiHa, and CaSki), human hepatocellular carcinoma (HepG2 and Hep3B), and human myeloid leukemia (K562, U937, and RAJI). The results showed that the leaf methanolic extracts of *U. longipes*, *Dasymaschalon* sp., *A. burmanicus,* and *M. modestum* induced apoptotic cell death in dose-dependent and cell-type dependent manner. Moreover, the leaf methanolic extract of *M. modestum* chosen for cell cycle analysis induced accumulation of cells in the subG1 phase, reflecting apoptotic cell death population. This effect was also dose-dependent and cell-type dependent.

Our results imply that these crude extracts might have some active compounds that function against human cancer cell lines. In this regard, all crude extracts were subsequently screened for certain chemical components such as alkaloids, flavonoids, etc. by phytochemical screening. Results from phytochemical screening showed that tannins and flavonoids were present in all the crude extracts. The leaf methanolic extracts of *U. longipes* and *Dasymaschalon* sp. contained saponins, whereas the leaf methanolic extracts from *U. longipes* and *A. burmanicus* contained alkaloids. All phytochemicals observed in our study have been previously reported about their anti-cancer activity in several studies. Flavonoids have the ability to induce apoptosis, block the cell cycle [35] by demolishing the structure of the spindle fiber [36], and inhibit angiogenesis [37]. Saponins are natural glycosides which have been previously proposed as anti-inflammatory, vaso-protective, hypocholesterolemic, antifungal, antiparasitic, and anti-cancer agents [38]. Alkaloids which can be isolated from plant sources [39] also have cytotoxicity and anti-cancer activity. For example, berberine can inhibit the proliferation of cancer cell lines by interfering with cell proliferation [40] and inducing apoptotic cell death [41]. Evodiamine or quinolone alkaloid showed anti-cancer activities by inducing cell cycle blocking in the K562 erythroleukemic cell line [42], causing DNA damage in MCF-7 breast cancer cells [43], inducing apoptosis in U937 human leukemic cells [44], interfering with angiogenesis [45], and interfering with cell metastasis in Lewis lung carcinoma (LLC) and B16-F10 melanoma [46].

When all the crude extracts were analyzed by HPLC, we found that rutin was an active compound in *U. longipes* and *Dasymaschalon* sp., and quercetin was an active compound in *U. longipes* and *M. Modestum*. These active compounds have been reported for numerous biological activities, such as anti-inflammatory, free radical scavenging, immunomodulatory, and cancer chemotherapy [28, 29]. Quercetin can promote the pro-apoptotic gene (Bax), enhance the anti-apoptotic gene (Bcl-2 and ERK) in leukemia cells, and activate caspase in osteosarcoma and oral cavity cancer cells [47, 48]. Rutin has been reported as having the ability to induce apoptosis in murine leukemia WEHI-3 cells in vitro and human leukemia HL-60 cells in vivo (murine xenograft model) [49]*.* To our knowledge, this is the first report showing rutin in leaf-derived crude extracts of *U. longipes* and *Dasymaschalon* sp., and quercetin in *U. longipes* and *M. modestum*. This finding suggests new sources for chemical compounds capable of inducing apoptotic cell death in vitro. However, these results are from preliminary screening of crude extracts; toxicity tests against normal cells, such as human mononuclear cells, hepatocytes etc., and an in-depth phytochemical analysis are necessary to guarantee the suitability of these extracts in therapeutic application against cancer.

## Conclusions

The crude extracts from leaves of *U. longipes, Dasymaschalon* sp., *A. burmanicus*, and *M. modestum* showed particular effects that were found to vary depending on the cancer cell lines examined. The leaves-derived crude extract of *M. Modestum* increased the percentage of the SubG1 phase in some cancer cell lines. Moreover, crude extracts from leaves of *U. longipes*, *Dasymaschalon* sp. and *M. modestum* provide a new source for rutin and quercetin, which might be capable of inducing cancer cell apoptotic death in a cell-type specific manner.
